# Asparaginase methylation and childhood obesity: cross-tissue patterns and parent–child concordance in a prospective cohort

**DOI:** 10.1007/s12519-026-01071-0

**Published:** 2026-09-15

**Authors:** Maria Niubó-Pallàs, Ariadna Gómez-Vilarrubla, Júlia Mollà-Martínez, Berta Mas-Pares, Alexandra Bonmatí-Santané, José-María Martínez-Calcerrada, Francis de Zegher, Lourdes Ibáñez, Abel López-Bermejo, Judit Bassols

**Affiliations:** 1 Maternal-Fetal Metabolic Research Group, Girona Institute for Biomedical Research (IDIBGI), Edifici M2 IAS, 17190 Salt, Spain; 2 Pediatric Endocrinology Research Group, Girona Institute for Biomedical Research (IDIBGI), 17190 Salt, Spain; 3 Department of Gynecology, Dr. Josep Trueta Hospital, 17007 Girona, Spain; 4 Department of Development and Regeneration, University of Leuven, 3000 Louvain, Belgium; 5 Endocrinology, Pediatric Research Institute, Sant Joan de Déu Children’s Hospital, 08950 Esplugues de Llobregat, Spain; 6 Centro de Investigación Biomédica en Red de Diabetes y Enfermedades Metabólicas Asociadas, Instituto de Salud Carlos III, 28029 Madrid, Spain; 7 Department of Pediatrics, Dr. Josep Trueta Hospital, 17007 Girona, Spain; 8 Department of Medical Sciences, University of Girona, 17003 Girona, Spain

**Keywords:** DNA methylation, Epigenetics, Obesity, Pediatric, Placenta

## Abstract

**Background:**

Early-life epigenetic mechanisms may contribute to the developmental programming of childhood obesity. We aimed to examine whether placental asparaginase (*ASPG*) DNA methylation (DNAm) is associated with adiposity at 6 years of age and to explore its cross-tissue patterns and parent–child concordance.

**Methods:**

In a prospective birth cohort, placental *ASPG* DNAm was quantified by pyrosequencing and related to anthropometric and cardiometabolic outcomes in children at 6 years (*n* = 180). DNAm was also measured in child peripheral blood (*n* = 127) and in maternal and paternal blood samples during pregnancy (*n* = 44).

**Results:**

Increased placental *ASPG* DNAm was associated with increased adiposity and cardiometabolic markers at 6 years and increased odds of overweight/obesity [odds ratio (OR) = 1.06, 95% confidence interval (CI) = 1.01–1.11, *P* = 0.01]. Similar associations were observed for child blood (OR = 1.19, 95% CI = 1.04–1.36, *P* = 0.01), although these analyses were cross-sectional. Placental and child DNAm levels were positively correlated (*r* = 0.40, *P* < 0.01), and maternal–child DNAm was concordant (*r* = 0.48, *P* < 0.01), although the parental analyses were exploratory and based on a limited sample size (*n* = 44).

**Conclusions:**

*ASPG* DNAm is associated with childhood adiposity and shows cross-tissue and parent–child concordance, suggesting its potential role as an early-life epigenetic marker associated with metabolic risk. Given the modest effect sizes and exploratory nature of some analyses, these findings warrant cautious interpretation and require replication in independent cohorts.

**Graphical Abstract:**

**Figure:**
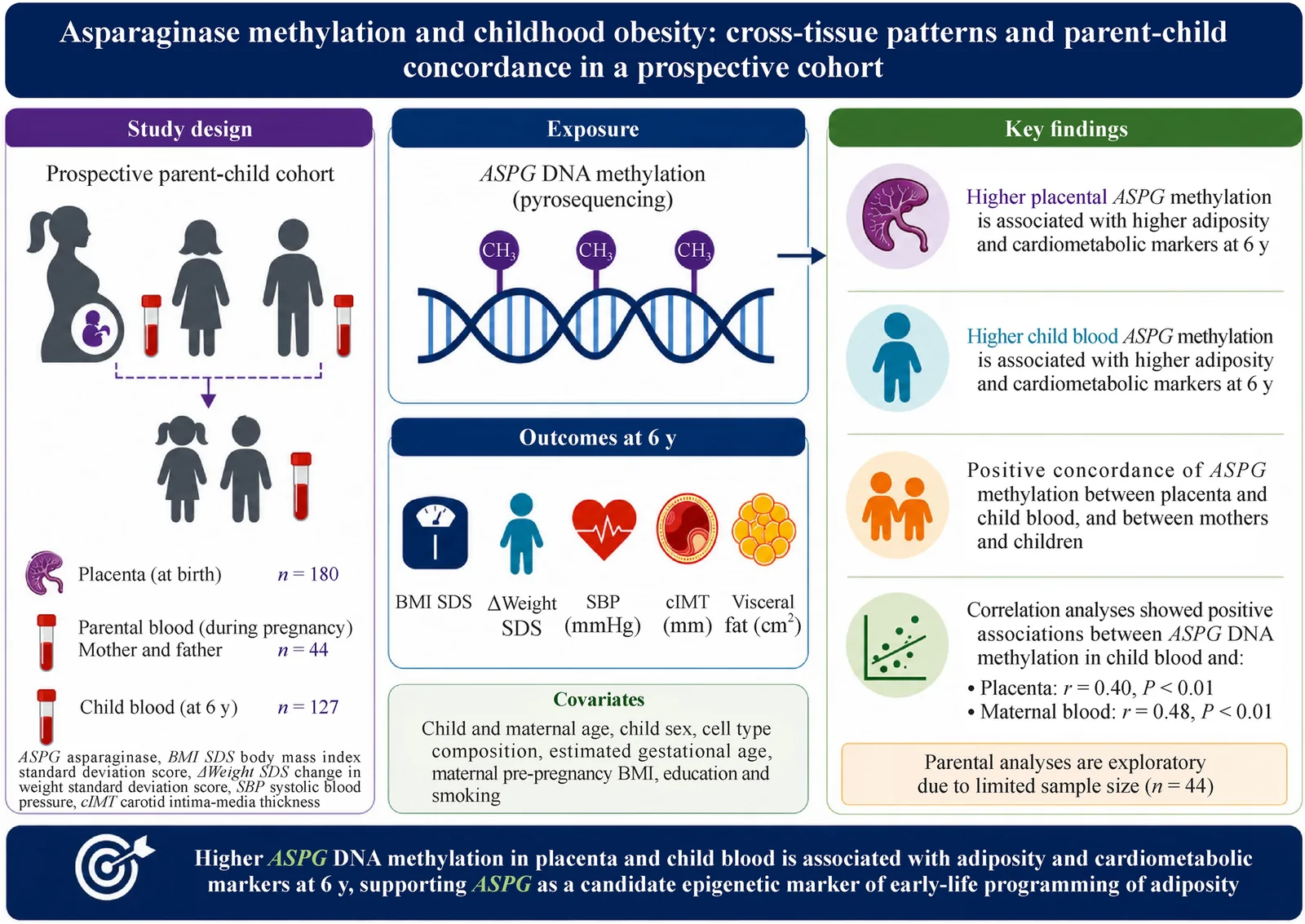

**Supplementary Information:**

The online version contains supplementary material available at https://doi.org/10.1007/s12519-026-01071-0.

## Introduction

The global prevalence of childhood obesity has increased dramatically in recent decades, representing a major public health concern with long-term metabolic, cardiovascular, and psychosocial consequences [1]. Mounting evidence supports the notion that susceptibility to obesity and related disorders arises not only from postnatal lifestyle factors but also from biological mechanisms involved in fetal development. According to the Developmental Origins of Health and Disease framework, intrauterine exposure can induce persistent molecular alterations that shape growth, energy metabolism, and the regulation of adiposity throughout life [2].

Among these molecular mechanisms, DNA methylation (DNAm) has emerged as a key mediator of developmental epigenetic programming [3]. DNAm involves the addition of methyl groups to cytosine residues at CpG sites, thereby regulating gene expression without altering the underlying DNA sequence. It plays essential roles in genomic stability, imprinting, and cell differentiation and is particularly dynamic during early embryogenesis and placental development. Environmental and nutritional factors can alter these methylation patterns during critical windows, leading to long-lasting effects on metabolic regulation and disease susceptibility, including obesity and insulin resistance [4].

The placenta, as the critical interface between the maternal and fetal environments, plays a central role in modulating nutrient, hormonal, and immune signals that influence fetal growth trajectories [5]. Placental epigenetic modifications are thought to reflect adaptive responses to these environmental cues. Several epigenome-wide association studies (EWAS) have identified placental loci whose DNAm levels correlate with metabolic outcomes in offspring [6–9]. These findings highlight the placenta as both a biosensor and a potential effector of early-life metabolic programming. However, whether DNAm markers established at birth persist over time and across tissues remains unknown.

Asparaginase (*ASPG*) has emerged as a biomarker of interest for health. It has long been used as a therapeutic enzyme in the treatment of acute lymphoblastic leukemia, where it depletes circulating asparagine and selectively inhibits the growth of tumor cells that depend on extracellular asparagine for survival [10]. Beyond its antineoplastic action, the activity of *ASPG* may be of interest for obesity and insulin resistance, as fluctuations in asparagine levels can modulate key signaling pathways, such as mechanistic target of rapamycin, AMP-activated protein kinase, and sterol regulatory element-binding protein 1, which govern lipid and glucose metabolism [11, 12]. Experimental studies in mice have indicated that hepatic *ASPG* expression is induced under metabolic stress, promoting lipid accumulation, inflammation, and insulin resistance [13, 14]. Conversely, the modulation of asparagine availability influences adipogenesis and systemic lipid metabolism [15]. Consistent with these animal data, recent human studies have demonstrated that hepatic *ASPG* negatively regulates insulin signaling [14] and that elevated circulating asparagine levels are associated with metabolic syndrome, obesity and the risk of type 2 diabetes [16–19].

Emerging evidence suggests that certain epigenetic signatures, particularly those established during pregnancy, may be mirrored in postnatal blood, reflecting coordinated and systemic developmental regulation [20, 21]. Moreover, parental germline and somatic epigenetic states are associated with metabolic traits in offspring, suggesting that specific methylation patterns can be inherited across generations in response to shared environmental and nutritional exposures [22, 23].

The present study aimed to examine the association between placental *ASPG* DNAm and offspring adiposity at the age of 6 years in a prospective birth cohort. Beyond this, we propose a developmental hypothesis whereby the prenatal environment influences *ASPG* DNAm patterns in the placenta, which may persist across tissues into postnatal life and contribute to the regulation of childhood adiposity. To address this, we evaluated the cross-tissue concordance of *ASPG* DNAm between placental and child blood and explored parent–child concordance to assess shared early-life influences.

## Methods

### Study population

We used data from the Girona Prenatal Study (EPG cohort, Estudi Prenatal Girona), a population-based prospective cohort established in Girona (Spain). The cohort comprises extensive biological, clinical, and perinatal information from families recruited during pregnancy, along with placental samples collected at delivery and child follow-up data at 6 years of age. Pregnant women were recruited during routine antenatal visits, and both maternal and paternal blood samples were obtained during the second trimester of pregnancy. Placental tissue was collected at birth, and the children were reassessed at the age of 6 years, when clinical measurements and peripheral blood samples were obtained.

### Inclusion and exclusion criteria

Eligible participants met the following inclusion criteria: Caucasian origin, singleton pregnancy, term delivery (37–40 gestational weeks), and written informed consent from both parents. The exclusion criteria were maternal medical, surgical, or obstetric complications (including pregestational or gestational diabetes, hypertension, preeclampsia or glucose intolerance), fetal growth restriction, neonatal malformations or asphyxia, assisted reproductive technologies, and maternal alcohol or drug abuse during pregnancy. The study protocol was approved by the Institutional Review Board of Dr. Josep Trueta Hospital (Girona).

### Perinatal data for mothers, fathers, and newborns

Information on pregnancy, labor, and delivery was retrieved from standardized medical records. Maternal weight at 6–9 weeks of gestation was used as a proxy for pregestational weight, and pregestational body mass index (BMI) was calculated as weight divided by height squared (kg/m^2^). Paternal height and weight were self-reported, and paternal BMI was calculated using the same formula. Newborn anthropometry was assessed immediately after birth using a calibrated scale and measuring board. Sex- and gestational age-adjusted *Z* scores [standard deviation score (SDS)] for birth weight and length were derived from regional standards.

### Clinical and anthropometric assessments for children

At the 6-year follow-up visit, weight was measured in light clothing using a calibrated scale, and height was measured without shoes using a Harpenden stadiometer. BMI and corresponding age- and sex-adjusted *Z* scores were calculated as described above. Waist circumference was measured in the supine position at the umbilical level, and hip circumference was measured while the greater trochanters were standing. Changes between birth weight and weight at 6 years (Δweight-SDS) were calculated as the difference between weight-SDS at 6 years and birth weight-SDS. Blood pressure [systolic blood pressure (SBP) and diastolic blood pressure] was assessed using an oscillometric device and measured on the right arm after 10 minutes of rest in the supine position. The mean of two measurements was used in the analyses.

Ultrasound measurements were performed using validated protocols previously described by our group [24]. Briefly, children were examined in the supine position, and all scans were obtained by the same trained operator using a high-resolution ultrasound system (MyLab™ 25, Esaote, Firenze, Italy) equipped with a 7.5–12 MHz linear transducer. Subcutaneous and visceral fat thickness were measured at the subxiphoid level, and carotid intima–media thickness (cIMT) was assessed at the right distal common carotid artery, approximately 1 cm proximal to the carotid bifurcation. The mean of three measurements was used in the analyses.

Fasting blood samples were collected after an overnight fast. Serum glucose levels were measured using the hexokinase method. Insulin was quantified by immunochemiluminescence (IMMULITE 2000, Diagnostic Products), with a detection limit of 0.4 mIU/L and intra- and inter-assay coefficients of variation (CVs) < 10%. Insulin resistance was estimated using homeostatic model assessment for insulin resistance (HOMA-IR): HOMA-IR = fasting insulin (mIU/L) × fasting glucose (mM)/22.5. Serum triglyceride levels were measured using glycerol-phosphate oxidase (ARCHITECT, Abbott Laboratories), with a detection limit of 5 mg/dL and CVs < 5%. High-density lipoprotein-cholesterol was quantified by a homogeneous selective detergent method (ARCHITECT, Abbott Laboratories), with a detection limit of 2.5 mg/dL and CVs < 4%. Low-density lipoprotein (LDL)-cholesterol levels were calculated using the Friedewald formula.

### Tissue and blood sample collection

At birth, four placental biopsies (one per quadrant) were collected from the maternal side after removal of the decidua. The samples were subsequently washed with saline to remove blood and stored at – 80 °C. Fasting peripheral blood was collected from the children at 6 years of age and from the parents during pregnancy. The leukocyte fraction was isolated by centrifugation and stored at – 80 °C.

### Study design

Genome-wide DNAm profiling was previously performed on 24 placental samples from the EPG cohort, as previously described [6]. Using these data, we identified a CpG site within the *ASPG* gene associated with offspring BMI-SDS at 6 years. In the present study, we focused on this CpG site for further validation and intergenerational analyses.

**Fig. 1:**
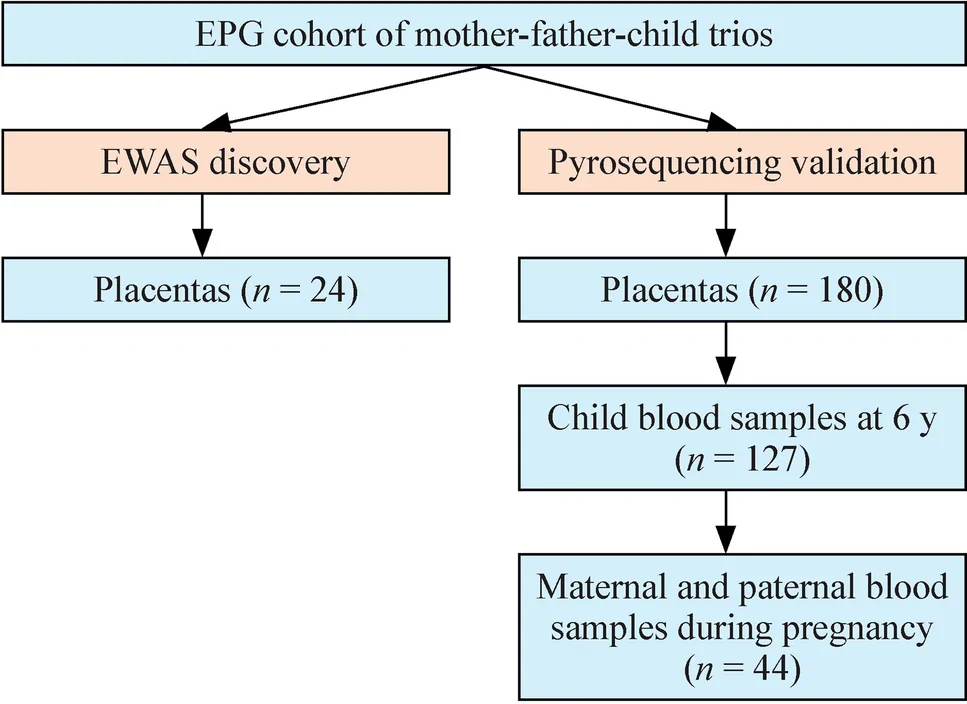
Flow diagram illustrating the study design and sample distribution across tissues and participants. *EPG* Girona Prenatal Study, *EWAS* epigenome-wide association studies

*ASPG* DNAm was validated by pyrosequencing in an independent set of 180 placentas. These validation samples were selected to ensure comparability with the EWAS subset in terms of key maternal and offspring characteristics (maternal age, maternal pre-pregnancy BMI and child sex), thereby reducing potential selection bias and maximizing methodological consistency. Among these 180 placentas, 127 had corresponding child peripheral blood samples available at the age of 6 years, and 44 additionally had maternal and paternal blood samples collected during pregnancy, enabling the assessment of inter-tissue and intergenerational *ASPG* DNAm patterns. The overall study design and sample flow are presented in Fig. 1.

### Placental DNA methylation analyses

Genome-wide DNAm profiling was performed using the Infinium® Human MethylationEPIC BeadChip (Illumina) as previously reported [6]. Briefly, raw IDAT files were processed using functional normalization implemented in the *minfi* R package (version 1.28.0). For each CpG site, *β* values (methylation level) and detection *P* values were extracted. Probes with a *P* value > 0.01, single-nucleotide polymorphism-associated probes, and probes on sex chromosomes were excluded. Differentially methylated CpG sites associated with BMI-SDS were identified using beta regression models (*betareg* package, version 2.0–13), with methylation level as the response and BMI-SDS as the predictor. Multiple testing was controlled using the false discovery rate. Adjusted *P* values < 0.05 were considered to indicate statistical significance. Methylation data are available at the Gene Expression Omnibus under accession number GSE192812.

### Pyrosequencing analyses

DNA extraction and pyrosequencing were performed following standard protocols as previously reported [6]. The CpG site analyzed (cg20965743) is located within the gene body of the *ASPG* gene and within a CpG island (chr14:104,552,012–104552478), according to the Illumina EPIC array annotation. Pyrosequencing assays were designed specifically to quantify DNAm at the same CpG site identified in the EWAS analysis. Briefly, genomic DNA from placental tissue and from the leukocytes of children (6 years) and parents (during pregnancy) was extracted using a QIAamp DNA Mini Kit (Qiagen, Germany). DNA bisulfite conversion was carried out with the EpiTect Fast DNA Bisulfite Kit (Qiagen). Bisulfite-converted DNA was amplified by real-time polymerase chain reaction using primers designed with PyroMark Assay Design 2.0 (Qiagen) (Supplementary Table 1). Pyrosequencing was performed on a PyroMark Q48 system (Qiagen), and methylation levels were quantified using PyroMark Q48 Autoprep software V2.4.2 (Qiagen).

### Statistical analysis

Statistical analyses were performed using SPSS version 22.0 (IBM Corp., NY, USA). The results are presented as the mean ± standard error of the mean. Continuous variables were assessed for normality using the Shapiro–Wilk test, and non-normally distributed variables were log-transformed. Comparisons between groups were performed using unpaired *t* tests. Associations between *ASPG* DNAm and child outcomes at 6 years were initially evaluated using Pearson correlation analyses, including cross-tissue and parent–child correlations. Multiple linear regression models were subsequently applied to assess the independent associations between *ASPG* DNAm and child outcomes, adjusting for potential confounders, including child age, sex, maternal pre-pregnancy BMI, gestational age and birth weight. Logistic regression models were used to examine the association between *ASPG* DNAm and the risk of overweight/obesity at 6 years, with the results expressed as odds ratios (ORs) and 95% confidence intervals (CIs). To facilitate interpretation, odds ratios were also expressed across the interquartile range of DNAm (75th vs. 25th percentile). Models were adjusted for the same covariates. Analyses were conducted separately for placental DNAm (*n* = 180), child peripheral blood DNAm (*n* = 127), and parental DNAm (*n* = 44), according to data availability. Given the exploratory nature and limited sample size of the parental analyses (*n* = 44), these findings should be interpreted with caution, as the study may be underpowered to detect small effect sizes. In the validation phase, analyses were conducted in a hypothesis-driven manner, focusing on a candidate CpG identified in the EWAS. Given this targeted approach, no formal multiple-testing correction was applied across the different child outcomes. Sensitivity analyses were performed by additionally adjusting the models for maternal smoking during pregnancy, gestational weight gain, and maternal glucose levels where available. A two-sided *P* value ≤ 0.05 was considered to indicate statistical significance.

## Results

### Population characteristics and tissue-specific* ASPG* DNA methylation levels

The main characteristics of the study population are summarized in Table 1**.** Maternal and paternal demographic and anthropometric variables were comparable across cohorts. Offspring also had similar gestational ages and birth anthropometry results. At 6 years of age, children exhibited largely comparable anthropometric, metabolic, and cardiovascular profiles across subsets.

**Table 1:** Maternal, paternal, and offspring characteristics in the discovery (by EWAS), validation (by pyrosequencing), and in blood-subset samples

Variables	EWAS	Pyrosequencing		
	Discovery placenta (*n* = 24)	Validation placenta (*n* = 180)	Child blood subset^a^ (*n* = 127)	Parental blood subset^a^ (*n* = 44)
Maternal				
Age (y)	31 ± 1	31 ± 1	31 ± 1	31 ± 1
Height (cm)	164 ± 1	162 ± 1	163 ± 1	162 ± 1
Pregestational weight (kg)	68.5 ± 2.9	65.0 ± 0.9	66.4 ± 1.1	69.0 ± 2.1
Pregestational BMI (kg/m^2^)	25.2 ± 1.0	24.5 ± 0.3	24.8 ± 0.4	26.2 ± 0.6
Paternal				
Age (y)	33 ± 1	34 ± 1	34 ± 1	34 ± 1
Height (cm)	179 ± 1	176 ± 1	176 ± 1	175 ± 1
Weight (kg)	84.7 ± 1.7	82.3 ± 0.9	82.4 ± 1.1	81.0 ± 1.9
BMI (kg/m^2^)	26.3 ± 0.4	26.4 ± 0.2	26.3 ± 0.3	26.4 ± 0.5
Offspring at birth				
Sex (female%)	50			
Gestational age (wk)	40 ± 0.2	40 ± 0.1	40 ± 0.1	40 ± 0.1
Birth weight (kg)	3.4 ± 0.1	3.4 ± 0.2	3.4 ± 0.3	3.3 ± 0.7
Birth weight-SDS	0.3 ± 0.1	0.2 ± 0.1	0.3 ± 0.1	0.1 ± 0.1
Birth length (cm)	50.1 ± 0.2	49.6 ± 0.1	49.9 ± 0.1	49.5 ± 0.3
Birth length-SDS	0.07 ± 0.10	– 0.04 ± 0.10	0.05 ± 0.10	– 0.04 ± 0.10
Offspring at 6 y				
Age (y)	6.2 ± 0.1	6.0 ± 0.1	6.0 ± 0.1	6.2 ± 0.1
Weight (kg)	23.7 ± 1.0	22.4 ± 0.4	23.1 ± 0.4	23.9 ± 0.8
Weight SDS	0.22 ± 0.20	0.04 ± 0.10	0.16 ± 0.10	0.27 ± 0.20
Height (cm)	120 ± 1.0	116 ± 1.0	117 ± 0.1	118 ± 1.2
Height-SDS	0.58 ± 0.20	0.11 ± 0.10	0.22 ± 0.10	0.16 ± 0.20
BMI (kg/m^2^)	16.3 ± 0.3	16.4 ± 0.2	16.5 ± 0.2	16.9 ± 0.3
BMI-SDS	– 0.02 ± 0.10	– 0.01 ± 0.10	0.08 ± 0.10	0.25 ± 0.10
ΔWeight-SDS	– 0.18 ± 0.20	– 0.24 ± 0.10	– 0.20 ± 0.10	0.11 ± 0.10
Waist (cm)	57.1 ± 1.7	56.2 ± 0.5	56.9 ± 0.6	59.2 ± 1.3
Hip (cm)	61.1 ± 1.8	59.5 ± 0.6	60.6 ± 0.7	62.5 ± 1.3
SBP (mmHg)	96.9 ± 3.0	95.8 ± 0.9	97.1 ± 1.1	96.0 ± 1.4
DBP (mmHg)	57.1 ± 1.1	57.1 ± 0.6	57.5 ± 0.7	58.4 ± 1.3
HDL-cholesterol (mg/dL)	57.0 ± 2.7	56.0 ± 0.8	56.1 ± 0.9	55.3 ± 1.4
LDL-cholesterol (mg/dL)	96.1 ± 3.8	97.8 ± 1.7	97.8 ± 2.0	94.2 ± 2.7
Triglycerides (mg/dL)	49.5 ± 2.7	50.0 ± 1.2	50.3 ± 1.4	50.4 ± 2.4
Glucose (mg/dL)	85.0 ± 1.7	82.9 ± 0.5	83.2 ± 0.5	83.9 ± 1.1
Insulin (mIU/L)	6.2 ± 0.5	5.2 ± 0.1	5.2 ± 0.2	6.0 ± 0.3
HOMA-IR	1.3 ± 0.1	1.1 ± 0.1	1.1 ± 0.1	1.2 ± 0.1
Subcutaneous fat (cm)	0.41 ± 0.03	0.39 ± 0.06	0.46 ± 0.02	0.52 ± 0.03
Visceral fat (cm)	0.45 ± 0.04	0.47 ± 0.07	0.47 ± 0.01	0.51 ± 0.03
cIMT (mm)	0.04 ± 0.01	0.04 ± 0.01	0.04 ± 0.01	0.04 ± 0.01

Data are presented as mean ± standard error of the mean. *EWAS* epigenome-wide association studies, *BMI* body mass index, *SDS* standard deviation score, *∆weigth-SDS* change in weight-SDS from birth to 6 years, *SBP* systolic blood pressure, *DBP* diastolic blood pressure, *HDL* high density lipoprotein, *HOMA-IR* homeostatic model assessment for insulin resistance, *cIMT* carotid intima-media thickness. ^a^The child blood subset (*n* = 127) and the parental blood subset (*n* = 44) are derived from the validation placenta sample (*n* = 180)

**Fig. 2:**
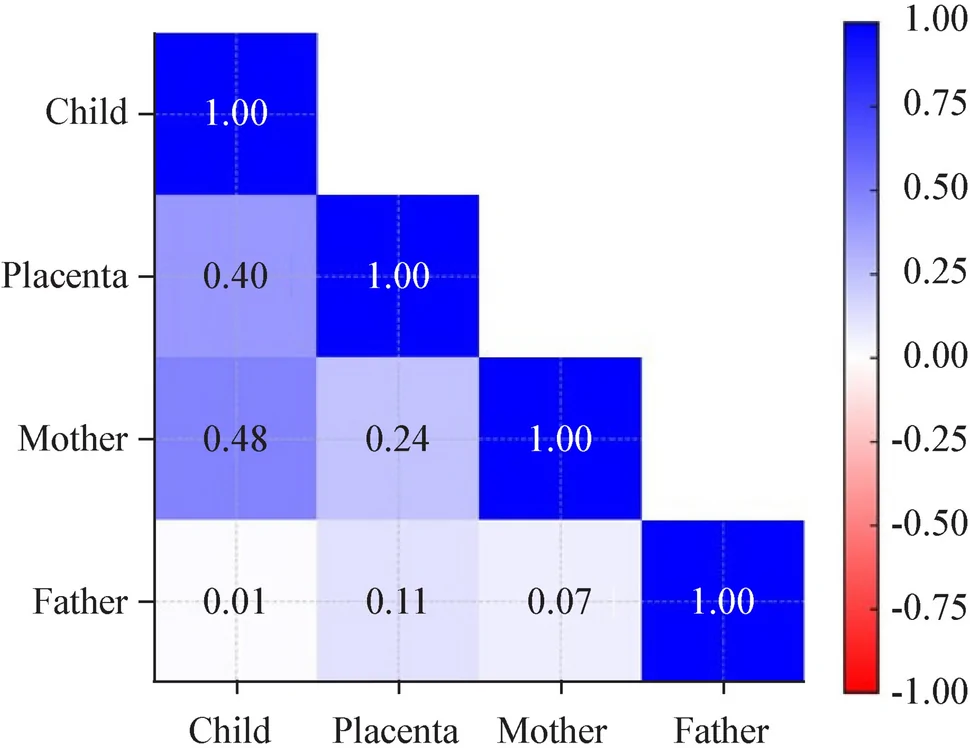
Heatmap showing cross-tissue correlations of *ASPG* DNA methylation in the placenta and in blood samples from mothers, fathers, and children

*ASPG* DNAm levels were similar across tissues and sample types, although the expected inter-tissue variability ranged from 11.1 ± 0.7 in the validation placental samples and 13.5 ± 2.8 in the EWAS placenta to 14.8 ± 0.3 in child blood, 15.4 ± 0.6 in maternal blood, and 17.3 ± 0.9 in paternal blood. Correlation analyses revealed positive associations between *ASPG* DNAm in child blood and both placenta (*r* = 0.40, 95% CI = 0.24–0.54, *P* < 0.01) and maternal blood (*r* = 0.48, 95% CI = 0.20–0.68, *P* < 0.01). In contrast, paternal blood methylation was not correlated with any other tissue (*r* ≤ 0.11, *P* > 0.05) (Fig. 2). Analyses involving parental methylation (*n* = 44) should be interpreted as exploratory given the limited sample size.

### Associations of *ASPG* DNA methylation with childhood adiposity and cardiometabolic parameters

**Fig. 3:**
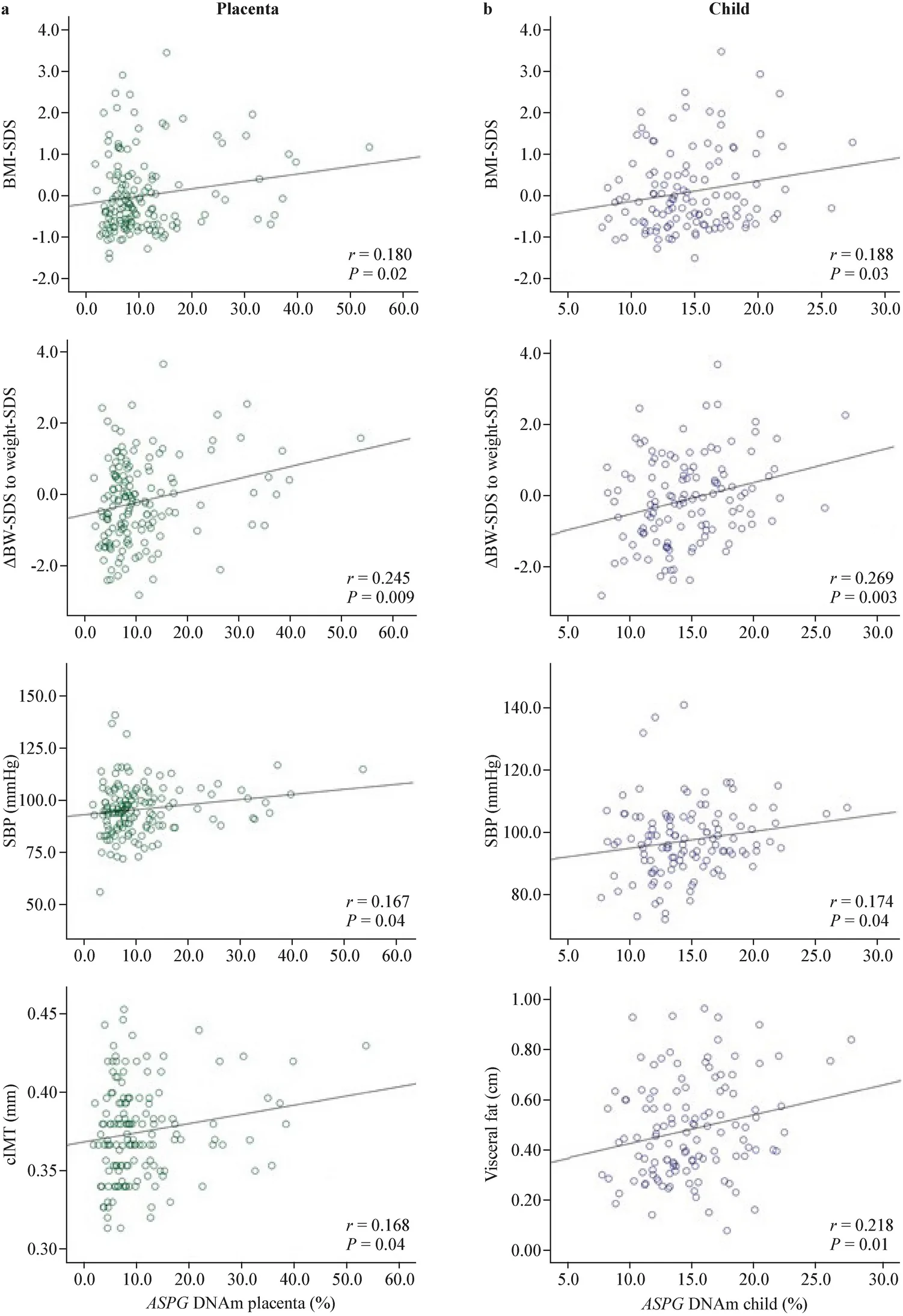
Correlations between *ASPG* DNAm and adiposity and cardiometabolic markers at age 6 years in (**a**) placenta and (**b**) child blood. *ASPG* asparaginase, *DNAm* DNA methylation, *BMI* body mass index, *BW* birthweight, *SBP* systolic blood pressure, *cIMT* carotid intima–media thickness, *SDS* standard deviation score

The pyrosequencing results validated the EWAS findings, as placental *ASPG* DNAm was positively associated with BMI-related measures at 6 years of age (weight-SDS: *r* = 0.180; BMI: *r* = 0.155; BMI-SDS: *r* = 0.165; all *P* < 0.05). In addition, placental *ASPG* DNAm correlated with growth-related and cardiometabolic parameters, including longitudinal changes in weight-SDS from birth to 6 years (Δweight-SDS: *r* = 0.245, *P* < 0.01), SBP (*r* = 0.167, *P* < 0.05), and cIMT (*r* = 0.168, *P* < 0.05). All these associations remained significant after adjustment for child age, sex, maternal pre-pregnancy BMI, gestational age, and birth weight (Table 2 and Fig. 3a).

**Table 2:** Correlation between *ASPG* methylation and child parameters at 6 years of age

Variables	*ASPG* placenta (*n* = 180)		*ASPG* child (*n* = 127)	
	*r* (95% CI)	*P*	*r* (95% CI)	*P*
Weight	0.150 (0.000–0.290)	NS	0.158 (– 0.020–0.320)	NS
Weight-SDS	**0.180 (0.030–0.320)**	**0.020**	0.170 (– 0.010–0.340)	NS
BMI	**0.155 (0.010–0.300)**	**0.040**	**0.192 (0.020–0.350)**	**0.030**
BMI-SDS	**0.165 (0.020–0.310)**	**0.040**	**0.188 (0.010–0.350)**	**0.030**
ΔWeight-SDS	**0.245 (0.100–0.380)**	**0.009**	**0.269 (0.010–0.420)**	**0.003**
Hip	0.120 (– 0.030–0.260)	NS	**0.181 (0.010–0.340)**	**0.040**
Waist	0.123 (– 0.020–0.270)	NS	**0.212 (0.040–0.340)**	**0.020**
SBP	**0.167 (0.020–0.310)**	**0.040**	0.174 (0.000–0.340)	0.040
Subcutaneous fat	0.125 (– 0.020–0.270)	NS	**0.232 (0.060–0.390)**	**0.010**
Visceral fat	0.039 (– 0.110–0.180)	NS	**0.218 (0.040–0.380)**	**0.010**
cIMT	**0.168 (0.020–0.310)**	**0.040**	0.103 (– 0.070–0.270)	NS
LDL-cholesterol	0.094 (– 0.050–0.240)	NS	**0.197 (0.020–0.360)**	**0.020**
Triglycerides	0.026 (– 0.120–0.170)	NS	**0.203 (0.030–0.360)**	**0.020**

Values are expressed as correlation coefficients (*r*) with 95% CIs and corresponding *P* values. In bold, values that are significant after adjusting for child age, sex, maternal pre-pregnancy BMI, gestational age, and birth weight are shown. *ASPG* asparaginase, *SDS* standard deviation score, *BMI* body mass index, *∆weigth-SDS* change in weight-SDS from birth to 6 years, *SBP* systolic blood pressure, *cIMT* carotid intima-media thickness, *CI* confidence interval, *NS* non-significant

*ASPG* DNAm measured in children’s blood showed a similar association pattern, with the strongest correlation observed for Δweight-SDS (*r* = 0.269, *P* < 0.01), followed by BMI and BMI-SDS (*r* = 0.192 and *r* = 0.188, respectively; both *P* < 0.05). Additional positive correlations were detected with measures of fat distribution, including waist circumference (*r* = 0.212, *P* < 0.05), hip circumference (*r* = 0.181, *P* < 0.05), subcutaneous fat (*r* = 0.232, *P* < 0.05), and visceral fat (*r* = 0.218, *P* < 0.05). Associations were also observed with cardiometabolic parameters such as SBP (*r* = 0.174, *P* < 0.05), LDL-cholesterol (*r* = 0.197, *P* < 0.05), and triglycerides (*r* = 0.203, *P* < 0.05). Most of these associations remained significant after adjustment for child age, sex, maternal pre-pregnancy BMI, gestational age, and birth weight (Table 2 and Fig. 3b). Although statistically significant, the observed correlations were modest in magnitude (r ranging from 0.15 to 0.27).

### Risk of childhood overweight/obesity associated with *ASPG* DNA methylation

Logistic regression models evaluating the association between *ASPG* DNAm and the risk of overweight/obesity at age 6 years adjusted for potential confounders (child age, sex, maternal pre-pregnancy BMI, gestational age and birth weight) revealed that higher placental *ASPG* DNAm was associated with a 6.0% increase in the odds of overweight/obesity (adjusted OR = 1.060, 95% CI = 1.013–1.109; *P* = 0.01). When individuals at the 75th versus the 25th percentile of DNAm were compared, the odds of overweight/obesity were approximately 1.47-fold greater. A stronger association was observed for *ASPG* DNAm in child blood (adjusted OR = 1.188, 95% CI = 1.040–1.357, *P* = 0.01), indicating an 18% increase in the odds of overweight/obesity. When individuals at the 75th versus the 25th percentile of DNAm were compared, the odds were approximately 2.4-fold greater. Additional adjustment for maternal smoking, gestational weight gain, and maternal glucose levels did not materially change the results, with similar or slightly stronger effect estimates observed. Although statistically significant, these effect sizes were modest, indicating relatively small increases in risk per unit change in DNAm.

## Discussion

Epigenetic variation in the placenta has previously been associated with childhood adiposity [6, 8, 9], although the persistence of these methylation patterns across tissues and their potential concordance within families remain poorly understood. In the present study, higher *ASPG* DNAm in both placental tissue at birth and child peripheral blood at 6 years of age was associated with greater adiposity and adverse cardiometabolic traits in childhood, including BMI-SDS, Δweight-SDS, blood pressure, and measures of fat distribution. In addition, *ASPG* DNAm in child blood correlated with methylation levels in the placenta and maternal blood, whereas no association was observed with paternal methylation. Together, these findings support *ASPG* DNAm as a candidate epigenetic marker associated with early-life metabolic programming.

*ASPG* is increasingly recognized as a metabolically relevant gene because of its role in asparagine-dependent pathways involved in lipid metabolism, adipogenesis, and insulin sensitivity [11, 12, 15]. Experimental studies have shown that *ASPG* expression is induced under metabolic stress and may contribute to lipid accumulation and insulin resistance [13, 14], whereas human metabolomic studies have linked circulating asparagine levels with obesity, metabolic syndrome, and type 2 diabetes risk [16–19]. Within this biological context, our findings are consistent with the potential role of *ASPG*-related pathways in metabolic regulation during early life. Importantly, the observed associations were consistent across placental and child blood samples, suggesting that *ASPG* DNAm may reflect broader developmental processes rather than tissue-specific alterations alone.

Previous studies have suggested that epigenetic marks may be shared across generations [25–28]. In our study, *ASPG* DNAm in maternal leukocytes during pregnancy was associated with methylation levels in children, whereas no association was observed between paternal and child methylation. Several, not mutually exclusive, mechanisms could explain this pattern. One possibility relates to differences in epigenetic reprogramming after fertilization, whereby paternal DNA undergoes predominantly active demethylation, while maternal DNA is mainly passively demethylated [29, 30]. These asymmetries could contribute to the differential continuity of methylation patterns between parents and offspring. Alternatively, the observed mother–child associations may reflect shared intrauterine exposures during pregnancy, which can influence maternal and fetal methylation patterns in parallel. Such pregnancy-related influences may recreate epigenetic marks in the fetus, resulting in similarities that mimic intergenerational patterns without the need for meiotic transmission [31]. Distinguishing between these explanations would require additional data; for instance, assessing maternal DNAm outside the gestational period could help determine whether the mother–child association persists independent of pregnancy-related physiological changes.

Cross-tissue comparisons offer additional context. Although maternal, paternal, and child methylation were measured in blood-derived leukocytes and placental tissue represents a biologically distinct matrix, *ASPG* DNAm in the placenta was correlated with child methylation, which was comparable in magnitude to the mother–child association. This is biologically consistent with the fetal origin of placental tissue, which shares a developmental lineage and intrauterine exposures with the fetus [32]. In contrast, maternal and placental methylation did not correlate, reflecting their distinct cellular origins and epigenetic programs. Thus, while placenta–child and mother–child associations may reflect shared developmental or environmental influences, the absence of a maternal–placental correlation underscores the epigenetic independence of these tissues [33]. Overall, these patterns are compatible with environmentally mediated similarities and potential parent–child epigenetic concordance, and future studies incorporating preconception parental samples and longitudinal methylation profiles will be needed to clarify these mechanisms.

Although the observed associations between *ASPG* DNAm and adiposity-related outcomes were statistically significant, their magnitude was modest. This finding is consistent with the multifactorial nature of obesity, where individual epigenetic marks are expected to contribute only a small proportion of the overall risk. This finding is also consistent with those of previous studies of placental epigenetic markers associated with childhood adiposity, where individual CpG loci generally explain only a limited proportion of phenotypic variability [6, 8, 9]. Therefore, *ASPG* DNAm should not be interpreted as a strong individual predictor but rather as a potential epigenetic marker reflecting early-life biological processes involved in metabolic programming. In practical terms, the observed odds ratios indicate relatively small increases in risk per unit change in DNAm, although differences across the distribution of DNAm may still translate into meaningful differences at the population level.

The strengths of this study include the use of a well-characterized prenatal cohort with prospectively collected biological samples from mothers, fathers, placental tissue, and children, allowing simultaneous assessment of cross-tissue continuity and parent–child concordance. The availability of detailed anthropometric, metabolic, and ultrasound-based adiposity measurements at 6 years of age further strengthens the interpretation of the findings. In addition, the combination of EWAS discovery and targeted validation in an independent sample enhances the robustness of the results.

Several limitations should also be acknowledged. First, the sample size, particularly for parental analyses, may have limited the statistical power to detect small effect sizes. Second, despite adjusting for key maternal and child covariates, residual confounding by environmental, socioeconomic, nutritional, or genetic factors cannot be excluded. Third, we measured DNAm at a single CpG and in heterogeneous cell populations and therefore could not determine whether the observed associations reflected cell type–specific changes or broader regulatory mechanisms. In addition, while associations involving placental DNAm and childhood outcomes were prospectively assessed, analyses involving DNAm in child blood at 6 years were cross-sectional, limiting causal inference and increasing the possibility of reverse causation or bidirectional relationships between DNAm and adiposity. Therefore, the interpretation of associations differs between tissues: placental DNAm may reflect early-life programming processes, whereas DNAm measured in child blood may represent concurrent or downstream biological changes associated with adiposity. Moreover, comparisons between placental tissue and peripheral blood should be interpreted with caution given their distinct cellular compositions and biological functions. Finally, although this study followed a hypothesis-driven candidate CpG approach, multiple-child outcomes were explored, which may increase the probability of chance findings. Therefore, these results should be interpreted with caution and warrant replication in independent cohorts.

In conclusion, our findings identify *ASPG* DNAm in both placenta and child blood as a consistent correlate of adiposity and overweight/obesity at 6 years of age, highlighting *ASPG* DNAm as a potential early-life epigenetic marker associated with metabolic risk. In addition to these phenotype–epigenotype associations, *ASPG* DNAm showed cross-tissue continuity from the placenta to child blood and parent–child concordance, particularly between mothers and children. These patterns are compatible with environmentally mediated similarities and early-life influences. To our knowledge, this is the first human study to examine *ASPG* DNAm across placental tissue, child blood, and parental blood in relation to childhood adiposity. However, the predictive value of *ASPG* DNAm was not formally assessed in this study, and future work incorporating discrimination and calibration metrics is needed to evaluate its potential clinical utility. Future studies incorporating multigenerational sampling and functional assays in metabolically relevant tissues will help clarify the biological relevance of *ASPG* DNAm and its potential role in metabolic programming.

## Supplementary Information

Below is the link to the electronic supplementary material.

Supplementary file 1 (PDF 8 KB)

## Data Availability

The DNA methylation datasets generated and analyzed during the current study are publicly available in the Gene Expression Omnibus (GEO) repository under accession number GSE192812. Additional data supporting the findings of this study are available from the corresponding author upon reasonable request.
