# Supplementary material for: Asparaginase methylation and childhood obesity: cross-tissue patterns and parent–child concordance in a prospective cohort
Source: World J Pediatr. 2026 Sep 15;22(8):899–909. doi: 10.1007/s12519-026-01071-0 (PMC13615054; doi:10.1007/s12519-026-01071-0)
Supplement: Supplementary file 1 [file 12519_2026_1071_MOESM1_ESM.pdf]

Supplementary Table 1 Pyrosequencing primers design

**Gene**		**Primers**	**Metling temperature (ºC)**	**Amplicon lenght (bp)**
ASPG	Fw	ATTGGTATGAGGAGTGAGTT	53.4	
	Rv	**BIOT-** AACCCACCCCAATCTCCTCTATC	58.1	262
	Seq	GGTGAGTTAGAGATTTTGGG	46.9	

Fw forward primer, *Rv* reverse primer, *Seq* sequence primer, *bp* base pairs
